# Exploring the Ability of Electronic Nose Technology to Recognize Interstitial Lung Diseases (ILD) by Non-Invasive Breath Screening of Exhaled Volatile Compounds (VOC): A Pilot Study from the European IPF Registry (eurIPFreg) and Biobank

**DOI:** 10.3390/jcm8101698

**Published:** 2019-10-16

**Authors:** Ekaterina Krauss, Jana Haberer, Olga Maurer, Guillermo Barreto, Fotios Drakopanagiotakis, Maria Degen, Werner Seeger, Andreas Guenther

**Affiliations:** 1 Universities of Giessen and Marburg Lung Center (UGMLC), Member of the German Center for Lung Research (DZL), Klinikstr. 33, 35392 Giessen, Germany; ekaterina.krauss@innere.med.uni-giessen.de (E.K.); janazoelitz@gmail.com (J.H.); jutta.schlegel@innere.med.uni-giessen.de (O.M.); guillermo.barreto@mpi-bn.mpg.de (G.B.); fdrakopanagiotakis@gmail.com (F.D.); werner.seeger@innere.med.uni-giessen.de (W.S.); 2 European IPF Registry & Biobank (eurIPFreg), 35394 Giessen, Germany; m.degen@klinik-waldhof.de; 3 Lung Cancer Epigenetic, Max Planck Institute Bad Nauheim, Ludwigstraße 43, 61231 Bad Nauheim, Germany; 4 Agaplesion Lung Clinic Waldhof-Elgershausen, Gruener Weg, 35753 Greifenstein, Germany; 5 Cardio-Pulmonary Institute (CPI), Klinikstr. 33, 35392 Gießen, Germany; 6 Brain and Lung Epigenetics (BLUE), Laboratoire Croissance, Réparation et Régénération Tissulaires (CRRET), CNRS ERL 9215, Université Paris Est Créteil, F-94000 Créteil, France

**Keywords:** electronic Nose (eNose, Aeonose^®^), idiopathic pulmonary fibrosis (IPF), European Registry for Idiopathic Pulmonary Fibrosis (eurIPFreg), interstitial lung diseases (ILD), volatile organic compounds (VOC)

## Abstract

Background: There is an increasing interest in employing electronic nose technology in the diagnosis and monitoring of lung diseases. Interstitial lung diseases (ILD) are challenging in regard to setting an accurate diagnosis in a timely manner. Thus, there is a high unmet need in non-invasive diagnostic tests. This single-center explorative study aimed to evaluate the usefulness of electronic nose (Aeonose^®^) in the diagnosis of ILDs. Methods: Exhaled volatile organic compound (VOC) signatures were obtained by Aeonose^®^ in 174 ILD patients, 23 patients with chronic obstructive pulmonary disease (COPD), and 33 healthy controls (HC). Results: By dichotomous comparison of VOC’s between ILD, COPD, and HC, a discriminating algorithm was established. In addition, direct analyses between the ILD subgroups, e.g., cryptogenic organizing pneumonia (COP, *n* = 28), idiopathic pulmonary fibrosis (IPF, *n* = 51), and connective tissue disease-associated ILD (CTD-ILD, *n* = 25) were performed. Area under the Curve (AUC) and Matthews’s correlation coefficient (MCC) were used to interpret the data. In direct comparison of the different ILD subgroups to HC, the algorithms developed on the basis of the Aeonose^®^ signatures allowed safe separation between IPF vs. HC (AUC of 0.95, MCC of 0.73), COP vs. HC (AUC 0.89, MCC 0.67), and CTD-ILD vs. HC (AUC 0.90, MCC 0.69). Additionally, to a case-control study design, the breath patterns of ILD subgroups were compared to each other. Following this approach, the sensitivity and specificity showed a relevant drop, which results in a poorer performance of the algorithm to separate the different ILD subgroups (IPF vs. COP with MCC 0.49, IPF vs. CTD-ILD with MCC 0.55, and COP vs. CT-ILD with MCC 0.40). Conclusions: The Aeonose^®^ showed some potential in separating ILD subgroups from HC. Unfortunately, when applying the algorithm to distinguish ILD subgroups from each other, the device showed low specificity. We suggest that artificial intelligence or principle compound analysis-based studies of a much broader data set of patients with ILDs may be much better suited to train these devices.

## 1. Introduction

Interstitial lung diseases (ILD) comprise about 200 heterogeneous entities with lung fibrosis as a common trait [1]. The group is very diverse regarding etiology, therapy, and outcomes. Globally, the incidence of ILD and especially that of Idiopathic Pulmonary Fibrosis (IPF) is rising, which is associated with an economic healthcare burden [2]. The natural history of progressive ILD is characterized by a decline in lung function, worsening of symptoms and health-related quality of life, and early mortality, especially in familial forms [3,4]. Greater impairment in forced vital capacity (FVC) or diffusion capacity of the lungs for carbon monoxide (DLco), and a greater extent of fibrotic changes on a highly resoluted computed tomography scan (HRCT), are predictors of mortality in ILD patients [5,6]. 

However, the course of these diseases is heterogenous and cannot be predicted accurately for an individual patient. In some cases, e.g., IPF, the patient’s survival might be still limited despite novel antifibrotic therapies [7,8]. Although significant progress in the understanding of the pathogenesis of ILDs has been made, the natural course, progression factors, biomarkers, and the response to the treatment of an individual patient still cannot be reliably predicted [9,10,11]. 

Electronic noses (eNoses) are artificial sensor systems, usually consisting of a range of sensors for various chemicals of interest, which are able to detect patterns of volatile organic compounds (VOC) in exhaled breath and then use learned algorithms for classification of the ‘breath print’ and comparison with previously recorded samples [12]. The concept of the eNose is that metabolic and biochemical processes occurring in different diseases give rise to specific patterns of endogenous VOC, which results in a “volatolome” or a VOC signature. This could be evaluated by eNose’s chemical sensors, and serve as possible markers of some inflammatory, microbial, oxidative, and neoplastic conditions [11,13]. Applications of the eNose technology has already been implemented in the food and beverage industry, in monitoring air quality, as well as in the detection of explosive and chemical agents [14]. The recent gold standard still appears to be a gas chromatography-mass spectrometry (GC-MS), which has been proven to be a useful tool in a variety of applications [15].

The eNose used in our study (Aeonose^®^) is a compact, hand-held non-invasive electronic device, developed by the eNose Company (Zutphen, The Netherlands). The technique enables transferring calibration models and large-scale applications. Principally, VOC can only be recognized after a calibration phase, i.e., the device must be trained to learn a disease pattern. Furthermore, the database of breath prints that stores previous analyses has to be developed. Hence, new VOC would be matched with existing profiles through comparative pattern recognition analysis [16]. 

In a complementary approach, there are several other methods looking for specific compounds in exhaled air, e.g., multi-capillary column-ion mobility spectrometry or gas chromatography-mass spectrometry. In contrast to eNose, these methods are not based on pattern recognition techniques, since they are aimed at identifying individual molecules in exhaled breath instead of a unique composite VOC signal [17]. 

There is a high unmet clinical need to improve screening and to increase specificity of earlier ILD detection by adding a non-invasive reliable screening test. Because eNoses have been reported to identify patients affected by different types of respiratory diseases, they, therefore, might help establish an early ILD diagnosis to predict prognosis and response to the treatment [18]. Thus, as an easy-to-handle, non-invasive diagnostic tool, they could represent an important aid during the diagnostic process. 

To the best of our knowledge, there are no explorative studies published yet, in which eNoses have been used for ILD diagnosis.

## 2. Objectives of This Study

This prospective single-center, non-invasive explorative study is aimed at investigating the diagnostic accuracy of an Aeonose^®^ to distinguish different ILDs on the basis of VOC patterns.

## 3. Materials and Methods

### 3.1. Study Design and Data Collection

This explorative research was designed as a single-center, prospective, non-invasive study in subjects with ILD as well as HC and COPD patients as a second comparator group. The study cohort consisted of 174 consecutive ILD patients from the University of Giessen and Marburg Lung Center (UGMLC) sites in Giessen and Greifenstein, who were recruited in the European IPF Registry and Biobank (eurIPFreg and eurIPFbank). The eurIPFreg is as an Internet-based, multi-center registry interlinked with the European IPF Biobank (eurIPFbank, see also www.pulmonary-fibrosis.net), listed in ClinicalTrials.gov (NCT02951416), and approved by Ethics Committee of the Justus-Liebig-University of Giessen (111/08) [7]. 

The datasets used and analyzed during the current study are available from the corresponding author on reasonable request.

### 3.2. Subject Selection

Between 2013 and 2015, among a total of 174 incident and prevalent ILD patients from our outpatient ILD clinics in Giessen and Greifenstein, 23 COPD and 33 controls above 18 years of age were asked to participate and to provide written informed consent prior to inclusion. The diagnosis of each ILD patient was done, according to the recent ATS/ERS/JRS/ALAT Clinical Practice Guideline, and confirmed by the respective physician and by a centralized review of data (Andreas Guenther (AG), Fotious Drakopanagiotakis (FD), Maria Degen (MD)) [8]. Baseline characteristics of the ILD cohort from eurIPFreg are displayed in our previous publication [7]. The COPD patients and HC were taken as independent comparator groups. 

Healthy controls were volunteers who were largely clinic staff and students. Control subjects reported not to suffer from lung diseases or other chronic conditions and did not show abnormalities upon physical examination. 

Patients with COPD have a somewhat comparable smoking history and a similar age range as compared to ILD (at least IPF) subjects. Therefore, we chose to include them as another disease comparator. Included COPD subjects were recruited during a regular follow-up visit and were all in stages III and IV, exclusively. 

Exclusion criteria for eurIPFreg were: age under 18 years, missed informed content, and pregnancy. The additional exclusion criteria for this analysis were patients with known lung cancer. All patients were followed up the last time in April 2019.

### 3.3. Sample Collection and Data Analysis

All participants provided one exhaled-breath sample per patient by inhaling and exhaling for 5 min, by using a nose clamp, through the Aeonose^®^. The breath samples were provided at different time points regarding ILD and COPD diagnoses. The patients were not asked to withdraw from food or medication intake at the time point of the measurement, but were asked not to smoke 2 h prior to the measurement.

### 3.4. Statistical Analysis and Data Presentation

In the first phase, the device had to be trained in terms of pattern recognition. In this scenario, air composition was measured every 20 s. using two 32-step sinusoidal modulations of the sensor surface temperature. The main objective in the training phase was not to define a specific VOC in the measurement but rather to determine the pattern of resistance changes in the sensors caused by the absorptions of the various VOC’s in the breath of patients. This resulted in a graphic pattern specific for each disease.

After this, signatures of VOC of ILD patients were captured using the Aeonose^®^ and were compared to HC in prospective correlation analyses. Additionally, we performed direct analyses between the ILD subgroups to deeper validate the ability of disease-specific pattern recognitions. 

To evaluate VOC signatures, a software program called Aethena was used for pre-processing, data compression, and neural networking [19]. To interpret the Aeonose^®^ data, the following parameters were measured: the Area under the Curve (AUC), sensitivity, specificity, and Matthews’s correlation coefficient (MCC). 

The MCC is a measure of the quality of binary classifications and is generally regarded as a balanced measure that can be used even if the classes are of very different sizes. In essence, the MCC is a correlation between the observed and predicted binary classification, where a value of +1 represents a perfect condition, 0 represents no better than a random prediction, and −1 indicates total disagreement between the prediction and observation [20]. Comparisons between groups were performed using ROC-Analysis. 

All statistical procedures were performed using SPSS 24 (SPSS, IBM Corp). For baseline data, the summary descriptive statistic was generated with categorical data displayed as absolute numbers and relative frequencies. Continuous data were shown as mean (SD) for normally distributed data. Comparisons between groups were performed using a *t*-test.

### 3.5. Aeonose^®^ Data Presentation

In the presented graphs, the values predicted with the model are corrected by the threshold and displayed in the cor column. In this column (values on the Y-axis), a positive value means a positive prediction. The predicted values indicate how well the pattern of the unknown ‘predicted’ sample matches with the calculated sample, so, if there is a good fit, the value will be 1. If there is a bad fit, the value will be −1. The values on the X-axis represent measurement numbers, plotted from left to right, according to their chronological order. The area around the dotted line indicates the threshold in which there is uncertainty regarding the final attribution of a measurement. The Aeonose^®^ also applies a 10% band around the threshold, where every sample on the positive side is labelled as ‘likely positive’ and, on the negative side, as ‘likely negative’ [16]. 

## 4. Results

### Demographics

In the period between 2013 and 2015, a total of 174 ILD subjects were measured by Aeonose^®^ and divided into ILD subgroups, as shown below. The statistical analysis was performed in January 2018, and, after additional algorithm adjustments, the analysis was performed in April 2019. The demographic data and distribution of diagnoses are shown in Table 1. Table 2 shows the results of the group comparison.

The results of lung function and gas exchange data of CTD-ILD, COP, IPF, and COPD cohorts are presented in Table 3. 

**Figure 1:**
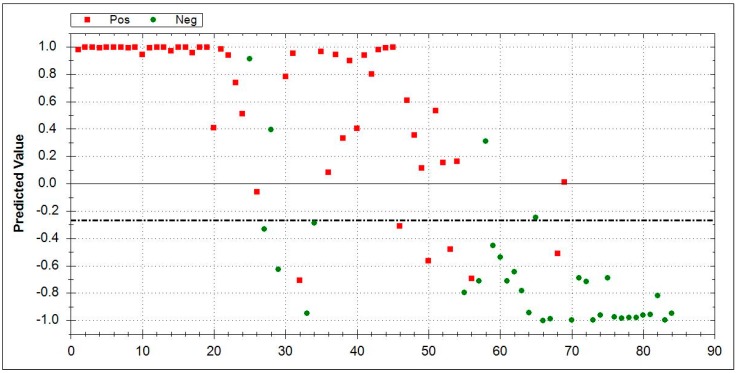
Direct comparison between idiopathic pulmonary fibrosis (*n* = 51, red squares) and HC (*n* = 33, green dots) by Aeonose^®^. IPF area 0–1: Red squares indicate correctly recognized IPF patients. Green dots denote false positive patients. HC area 0–−1: Green dots represent correctly identified healthy controls, and red squares mark false negative results. The dotted line is inserted for values around the threshold where there is doubt about which side it tends to, and, hence, reflects an area of uncertainty. Abbreviations: IPF-Idiopathic pulmonary fibrosis, HC- healthy controls.

In the first approach, the VOC patterns of IPF patients were directly compared to HC after a training (calibration) phase. The Aeonose^®^ was able to differentiate IPF-patients (*n* = 51) vs. HC (*n* = 33), which showed a sensitivity of 0.88, a specificity of 0.85, an AUC of 0.95, and an MCC of 0.73. Figure 1 displays the data. 

**Figure 2:**
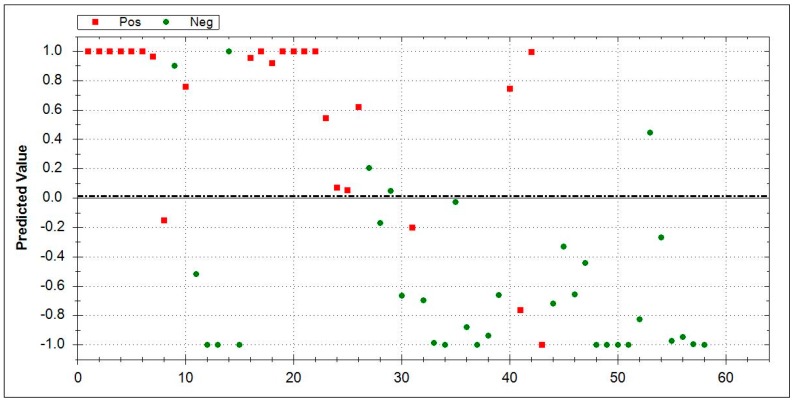
Direct comparison between CTD-ILD (*n* = 25, red squares) and HC (*n* = 33, green dots) by Aeonose^®^. CTD-ILD area 0–1: Red squares indicate correctly recognized CTD-ILD patients while green dots denote false positive patients. HC area 0–−1: Green dots represent correctly-identified healthy controls, while red squares mark false negative results. Abbreviations: CTD-ILD-connective-tissue diseases- associated ILD, HC- healthy controls.

By directly comparing patients with CTD-ILD (*n* = 25) vs. HC (*n* = 33), an AUC of 0.90, MCC of 0.69, sensitivity of 0.84, and specificity of 0.85 were encountered. Figure 2 shows the ability of Aeonose^®^ to identify CTD-ILD patients in direct comparison with HC.

**Figure 3:**
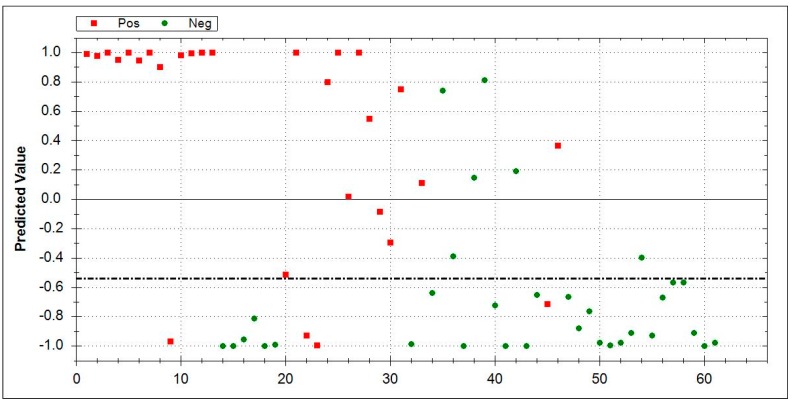
Direct comparison between COP (*n* = 28, red squares) vs. HC (*n* = 33, green dots). COP area 0–1: Red squares indicate correctly recognized COP patients while green dots denote false positive patients. HC area 0–−1: Green dots represent correctly identified healthy controls, while red squares mark false negative results. The dotted line is inserted for values around the threshold where there is doubt about which side it tends to, and, hence, reflects an area of uncertainty. Abbreviations: COP-cryptogenic organizing pneumonia, HC- healthy controls.

In a further direct comparison between cryptogenic organizing pneumonitis (COP, *n* = 28) vs. HC (*n* = 33), an AUC of 0.89 and MCC of 0.67 were obtained. Sensitivity was 0.86 and specificity was 0.82. Figure 3 summarizes the data.

Due to a limited sample size in other ILD subgroups, the further differentiation could not be safely performed by the Aeonose^®^ and was, therefore, left out. 

The direct comparison analyses forwarded promising and interesting results, with AUC as well as sensitivity and specificity values suitable for a potential use of the Aeonose^®^ as a diagnostic test. 

However, we had not checked the performance of the Aeonose^®^ in an independent, second control cohort. COPD patients were used in the analysis (*n* = 23). In comparison between COPD and HC, AUC 0.91, MCC 0.73, sensitivity 0.86, and specificity 0.88 were obtained. In direct assessment between COP (*n* = 28) and COPD, an AUC of 0.77, a MCC of 0.46, a sensitivity of 0.75, and a specificity of 0.71 were obtained. In the analysis of CTD-ILD (*n* = 25) vs. COPD (*n* = 23) Aeonose^®^ forwarded an AUC of 0.85, a sensitivity of 0.88, a specificity of 0.71, and an MCC of 0.61.

**Figure 4:**
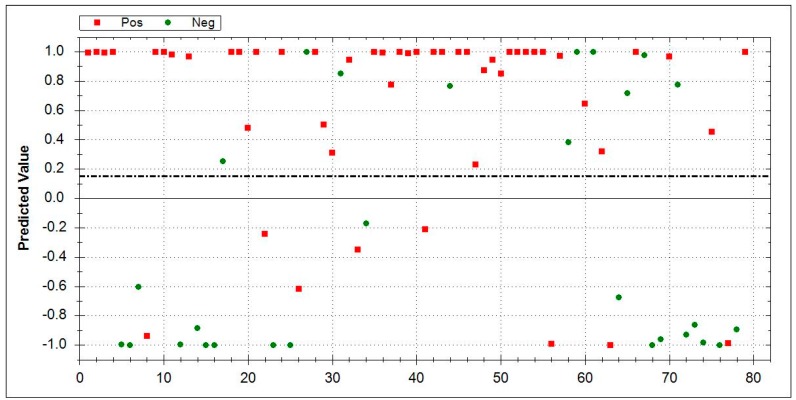
Direct comparison between IPF (*n* = 51, red squares) vs. COP (*n* = 28, green dots). IPF area 0–1: Red squares indicate correctly-recognized IPF patients. Green dots denote false positive patients. COP area 0–−1: Green dots represent correctly identified COP patients, while red squares mark false negative results. The dotted line is inserted around the threshold for uncertain cases and included two patients. Abbreviations: IPF-Idiopathic pulmonary fibrosis, COP-cryptogenic organizing pneumonia.

**Figure 5:**
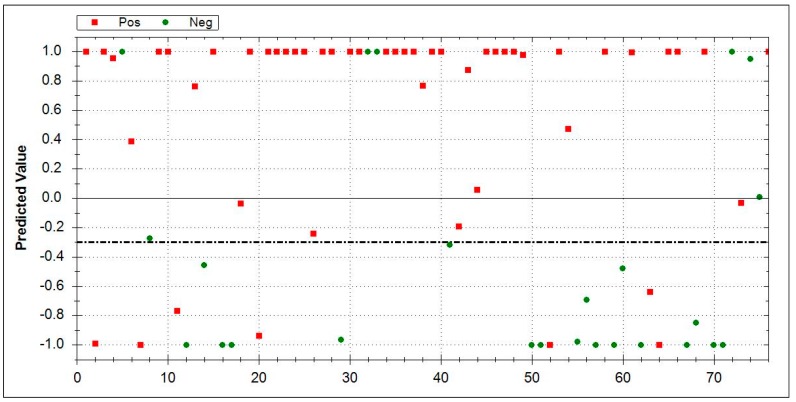
Direct comparison between IPF (*n* = 51, red squares) vs. CTD-ILD (*n* = 25, green dots). IPF area 0–1: Red squares indicate correctly recognized IPF patients. Green dots denote false positive patients. CT-ILD area 0–−1: Green dots represent correctly identified CTD-ILD, while red squares mark false negative results. The dotted line is inserted around the threshold for uncertain cases and included two patients. Abbreviations: IPF-Idiopathic pulmonary fibrosis, CTD-ILD-connective-tissue diseases- associated ILD.

**Figure 6:**
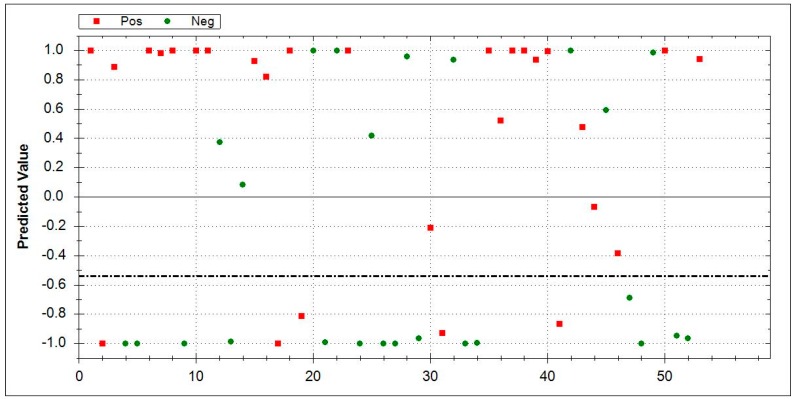
Direct comparison between COP (*n* = 28, red squares) vs. CTD-ILD (*n* = 25, green dots). Abbreviations: COP area 0–1: Red squares indicate correctly recognized COP patients. Green dots denote false positive patients. CTD-ILD area 0–−1: Green dots represent correctly identified CTD-ILD, while red squares mark false negative results. The dotted line is inserted around the threshold for uncertain cases and included two patients. Abbreviations: COP-cryptogenic organizing pneumonia, CTD-ILD-connective-tissue diseases- associated ILD.

To further validate the ability of eNose to recognize the disease-specific VOC pattern, we compared breath patterns of ILD subgroups to each other instead of applying a case-control study design. Following this approach, however, the sensitivity and specificity showed a relevant drop. Although the device was previously trained in disease-specific pattern recognition using two control cohorts (HC and COPD), Aeonose^®^ was only partly able to distinguish the groups correctly (Figure 4, Figure 5 and Figure 6).

By comparing ILD subgroups with each other, the Aeonose^®^ performed less accurately, whereas comparing the subgroups with HC, the device had good accuracy. In the group analysis between IPF (*n* = 51) vs. COP (*n* = 28), AUC of 0.82, sensitivity of 0.84, specificity of 0.64, and MCC of 0.49 were obtained. Figure 4 displays the results. 

In the analysis between IPF (*n* = 51) vs. CTD-ILD (*n* = 25), AUC of 0.84, sensitivity of 0.86, specificity of 0.68, and MCC of 0.55 were obtained. Figure 5 displays the results. 

In the analysis between COP (*n* = 28) vs. CTD-ILD (*n* = 25), an AUC of 0.75, a sensitivity of 0.82, a specificity of 0.56, and an MCC of 0.40 were obtained. Figure 6 shows the results. 

The results of the diagnostic performance of the Aeonose^®^ are summarized in Table 4. 

## 5. Discussion

The aim of this study was to investigate if Aeonose^®^ could be of diagnostic help in ILD’s recognition. We examined if ILD–specific VOC patterns can be clearly recognized by the Aeonose^®^ and distinguished from HC as well as pulmonary comorbidities such as COPD. After completing the training phase, we evaluated if Aeonose^®^ is able to reliably detect differences in the VOC pattern of IPF, COP, or CT-ILD. 

Without a doubt, our initial results reflecting the direct comparison of different ILD subgroups to HC and, following a case-control design as established in previous studies, were very appealing. The algorithm established in this study to separate ILD from controls and COPD patients resulted in good sensitivity and specificity in a case-control approach. In this regard, it appeared as if the Aeonose^®^ has some potential in recognizing ILD patients.

Nonetheless, knowing about the risk of bias due to the case-control design, as reviewed by Leopold et al., we extended our assessment from direct comparison to further correlations, by comparing subgroups within themselves [21]. In this case, although being previously trained in disease-specific pattern recognition, the Aeonose^®^ algorithm performed less effective and was not able to distinguish the breathome and to separate patients correctly. Instead, reduced MCC, sensitivity, and specificity values were encountered under these conditions. The ability of the Aeonose^®^ to safely separate these ILD entities from each other was noticeably lower and, therefore, cannot be offered for routine use. 

We suspect that the ILD subgroups could not be safely distinguished from each other due to the different possible reasons. One of them could be the sample size in the subgroup analysis, which leads to the possible insufficient training of the eNose. Another putative explanation lies in the training approach of the eNose, which is always based on a dichotomous comparison between two different conditions (e.g., ILD versus healthy controls). Such an approach does not allow for unsupervised clustering of data and safe attribution of volatile signatures to several conditions in parallel.

To this day, there are no known studies describing specific VOC patterns in fibrotic lung diseases. However, there are some successful publications with regard to a screening of various respiratory and systemic diseases, e.g., lung cancer, diabetes mellitus, or even evaluating VOC profiles in critically ill patients [22,23,24]. Without any doubt, the process of ILD diagnosis would profit from new non-invasive forms of diagnostics aside of imaging. Likewise, non-invasive prognostic and therapeutic markers are urgently needed. As an example, a comprehensive metabolome analysis could allow the tracking of metabolic pathways, and allow us to monitor the efficiency of therapeutic interventions [25]. Several molecules related to epithelial cell injury, matrix remodeling, and immune regulation have been discussed to be promising candidates [11]. 

In addition, a combination of multiple biomarkers may be useful to identify comprehensive individual signatures in ILD patients, which leads to a more personalized medicine [11]. Moreover, exhaled breath-based methods have been studied in the past decades for their applicability in the assessment of airway inflammation and as possible diagnostic tools in several inflammatory lung diseases, e.g., asthma or COPD [26,27]. In this case, a large number of biomarkers in breath have been examined as possible indicators of inflammation, to diagnose and monitor the diseases as well as to evaluate the response to treatment [28]. Therefore, exhaled breath analysis by means of eNose technology has been of great scientific interest over the last few years and is a rapidly emerging field of medicine. Still, despite all efforts, eNoses appear to not be ready for implementation as a medical diagnostic tool. 

Based on our results, we speculate that further large cohort, cross-sectional analyses are necessary to identify and validate the ILD subtype-specific VOC patterns, as well as to enhance the sensitivity and specificity of the Aeonose^®^ or any other electronic nose. Next to multi-variant analyses, one could also consider using artificial intelligence-based algorithms, and of unsupervised clustering of data, e.g., by the principle compound analysis currently employed in single cell omics.

### Study Limitations

This study analyzed a consecutive ILD cohort. Because it has not always been technically possible to take exhaled-breath samples precisely at the time point of diagnosis, the influence of the disease course could not be taken into account. Another study limitation could be that an advanced ILD could have different VOC profiles, as compared to the subjects with recently developed ILD, and that the differences in disease severity could influence VOC profiles as well. Although, VOC profiles are believed to be disease-specific, the still unknown influence of many diverse endogenous and exogenous cofounders (e.g., age, diet, alcohol consumption, or medication) is needed to take into account and to be evaluated in further eNose ILD studies. 

## 6. Conclusions

The algorithm developed in this study to separate ILD from controls and COPD patients using the Aeonose^®^ resulted in good sensitivity and specificity in separating these two conditions in a case-control approach. Unfortunately, when comparing the different ILD entities directly with each other, the performance of the Aeonose^®^ to safely separate these ILD entities from each other was markedly lower and is not offered for routine use.

Despite these somewhat disappointing results, we believe that VOC signatures, once being adequately clustered and annotated to the underlying pulmonary phenotype, may be used for rapid and safe differentiation of different ILD entities and to accomplish screening programs. We suggest that artificial intelligence or principal component analysis-based studies of a much broader data set of patients with ILDs may be much better suited to train these devices and, ultimately, to allow safe differentiation within ILDs.

## Figures and Tables

**Table 1:** Demographics of the eNose cohort, including ILD, COPD, and HC groups.

Group	Number	Mean Age at Baseline ± SD	Male		Smoking History		
	(*n*)	(years)	(*n*)	Current Smoker (*n*)	Ex-Smoker (*n*)	Never-Smoked (*n*)	Smoking History Unknown (*n*)
ILD	174						
CTD-ILD	25	66.4 ± 11.2	6	1	13	10	1
COP	28	67.2 ± 7.7	13	-	20	8	-
HP	20	63.2 ± 12.7	12	-	9	8	3
IPF	51	68.6 ± 8.3	37	2	33	15	1
Sarcoidosis	19	56.7 ± 14.3	9	2	6	11	-
uILD	20	65.5 ± 11.7	14	5	5	10	-
Asbestosis	5	72 ± 3.9	5	-	3	2	-
Other ILD (NSIP, RB-ILD, DIP)	6	66.8 ± 11.9	3	1	2	3	-
Healthy controls	33	34.4 ± 14.9	1	8	2	10	13
COPD	23	64.4 ± 9.4	18	2	17	2	2

Abbreviations: CTD-ILD-connective-tissue diseases- associated ILD, COP-cryptogenic organizing pneumonia, COPD-chronic obstructive pulmonary disease, HP-hypersensitivity pneumonitis, IPF-Idiopathic pulmonary fibrosis, uILD-unclassifiable ILD, NSIP-non-specific interstitial pneumonia, RB-ILD-respiratory bronchiolitis-associated ILD, DIP-desquamative interstitial pneumonia, *n*-number of patients, and SD-standard deviation.

**Table 2:** Results of group comparison (*t*-test).

	Significance (2-tailed)	Mean Difference	95% Confidence Interval (Lower)	95% Confidence Interval (Upper)
Mean Age at baseline	0.000	62.5200	Lower	Upper
Male	0.006	11.800	4.41	19.19
Ex-smoker (*n*)	0.007	11.000	3.86	18.14
Never-smoked (*n*)	0.000	7.900	4.82	10.98
Current smoker (*n*)	0.022	3.,000	.61	5..39

**Table 3:** The results of lung function and gas exchange data of CTD-ILD, COP, IPF, and COPD cohorts.

	CTD-ILD (*n* = 25)	COP (*n* = 28)	IPF (*n* = 51)	COPD (*n* = 23)
VC (% predicted), mean value ± SD	57.33 ± 6.51	87.38 ± 21.70	65.58 ± 17.46	87.00 ± 17.35
FVC (% predicted), mean value ± SD	50.67 ± 11.37	74.88 ± 24.89	57.33 ± 17.58	66.00 ± 23.52
FEV 1 (% predicted), mean value ± SD	52.67 ± 22.03	80.63 ± 30.31	62.13 ± 20.04	55.67 ± 18.01
DLCO (% predicted), mean value ± SD	49.67 ± 9.50	72.88 ± 14.87	56.71 ± 19.91	72.67 ± 25.82
pO2 (mm Hg) at rest, mean value ± SD	66.50 ± 13.94	74.42 ± 4.69	68.90 ± 9.07	65.03 ± 9.19
6MWD (meters), mean value ± SD	180 ± 158.74	386.25 ± 98.12	395.42 ± 106.65	320 ± 183.30

Abbreviations: FEV1-Forced expiratory volume, VC-Vital capacity, FVC-Forced vital capacity, DLCO-diffusing capacity of the lung for carbon monoxide, pO_2_-partial pressure of oxygen, 6MWD-six meters walking distance, CTD-ILD-connective-tissue diseases- associated ILD, COP-cryptogenic organizing pneumonia, COPD-chronic obstructive pulmonary disease, HP-hypersensitivity pneumonitis, IPF-Idiopathic pulmonary fibrosis.

**Table 4:** Diagnostic performance of the Aeonose^®^.

Groups	Number (n)	Sensitivity (%)	Specificity (%)	AUC	MCC
IPF vs. HC	51 vs. 33	0.88	0.85	0.95	0.73
CTD-ILD vs. HC	25 vs. 33	0.84	0.85	0.9	0.69
COP vs. HC	28 vs.33	0.86	0.82	0.89	0.67
COPD vs. HC	23 vs. 33	0.86	0.88	0.91	0.73
COP vs. COPD	28 vs. 23	0.75	0.71	0.77	0.46
CTD-ILD vs. COPD	25 vs. 23	0.88	0.71	0.85	0.61
IPF vs. COP	51 vs. 28	0.84	0.64	0.82	0.49
IPF vs. CTD-ILD	51 vs.25	0.86	0.64	0.84	0.55
COP vs. CTD-ILD	28 vs. 25	0.82	0.56	0.75	0.40

Abbreviations: AUC-area under the curve, CI-confidence interval, MCC-Matthews’s correlation coefficient, CTD-ILD-connective-tissue diseases- associated ILD, COP-cryptogenic organizing pneumonia, COPD-chronic obstructive pulmonary disease, and IPF-Idiopathic pulmonary fibrosis.

## Abbreviations

American Thoracic Society (ATS), European Respiratory Society (ERS), Japanese Respiratory Society (JRS), Latin American Thoracic Society (ALAT), Diffusing capacity of the lung for carbon monoxide (DLC0), Electronic nose (eNose), European IPF Registry (eurIPFreg), European IPF Biobank (eurIPFbank), Idiopathic pulmonary fibrosis (IPF), Interstitial lung diseases (ILD), Volatile organic compounds (VOC), CTD-ILD-connective-tissue diseases-associated ILD, COP-cryptogenic organizing pneumonia, HP-hypersensitivity pneumonitis, uILD-unclassifiable ILD, NSIP-non-specific interstitial pneumonia, RB-ILD-respiratory bronchiolitis-associated ILD, DIP-desquamative interstitial pneumonia, *n*-number of patients, SD-standard deviation.
